# An fMRI Investigation of Preparatory Set in the Human Cerebral Cortex and Superior Colliculus for Pro- and Anti-Saccades

**DOI:** 10.1371/journal.pone.0158337

**Published:** 2016-07-08

**Authors:** Michele Furlan, Andrew T. Smith, Robin Walker

**Affiliations:** Royal Holloway University of London, Egham Hill, Egham, Surrey, TW20 0EX, United Kingdom; Universidad de Chile, CHILE

## Abstract

Previous studies have identified several cortical regions that show larger BOLD responses during preparation and execution of anti-saccades than pro-saccades. We confirmed this finding with a greater BOLD response for anti-saccades than pro-saccades during the preparation phase in the FEF, IPS and DLPFC and in the FEF and IPS in the execution phase. We then applied multi-voxel pattern analysis (MVPA) to establish whether different neural populations are involved in the two types of saccade. Pro-saccades and anti-saccades were reliably decoded during saccade execution in all three cortical regions (FEF, DLPFC and IPS) and in IPS during saccade preparation. This indicates neural specialization, for programming the desired response depending on the task rule, in these regions. In a further study tailored for imaging the superior colliculus in the midbrain a similar magnitude BOLD response was observed for pro-saccades and anti-saccades and the two saccade types could not be decoded with MVPA. This was the case both for activity related to the preparation phase and also for that elicited during the execution phase. We conclude that separate cortical neural populations are involved in the task-specific programming of a saccade while in contrast, the SC has a role in response preparation but may be less involved in high-level, task-specific aspects of the control of saccades.

## Introduction

Under normal viewing conditions humans and non-human primates make saccadic eye movements in order to direct their gaze onto objects of interest for a period of relatively steady fixation for detailed visual analysis [1]. Saccadic eye movements are typically directed towards a peripheral stimulus (called a ‘pro-saccade’), but they can also be directed away from the stimulus on the basis of an instruction (an anti-saccade) as shown in the first studies by Hallett [2,3]. Both pro- and anti- saccades are under voluntary control [4], but performing an anti-saccade is more cognitively demanding than generating a pro-saccade. The correct generation of an anti-saccade involves the suppression of a response towards the peripheral stimulus (The so called visual-grasp reflex [5]) the translation of the desired goal and the generation of a saccade to the location opposite to the target onset [5]. Behaviourally, anti-saccades have longer latencies and are less accurate than equivalent pro-saccades and on some trials errors occur in which a saccade is directed towards the target [2,3,5]. Pro-saccade errors occur on some 10–20% of trials [5,6], although they decrease with practice and are often followed by secondary corrective saccade that can be of very short latency [2]. Patients with damage to frontal cortical regions [7–10] and those with psychopathological disorders such as schizophrenia [11,12] can show much higher error rates. The apparent simplicity of the anti-saccade task has made it a useful tool for investigating the underlying basis of higher-level cognitive control processes [5,6].

The involvement of frontal lobe structures in the control of anti-saccades has been supported by human functional brain imaging studies [13–17] in addition to clinical studies of brain-damaged patients [12]. Imaging studies have implicated a network of fronto-parietal regions including: dorsolateral prefrontal cortex (DLPFC), frontal eye fields (FEF), supplementary eye fields (SEF), anterior cingulate (AC), posterior parietal cortex (PPC), along with the thalamus and stratium subcortically [13,14]. Event-related functional magnetic resonance imaging (fMRI) has been applied to investigate the processes involved in generating pro- and anti-saccades. Connolly and colleagues [18] used fMRI to investigate the blood-oxygenated level dependent (BOLD) response during the preparatory period and showed an elevated response in the FEF for anti- compared to pro-saccades. This was interpreted as reflecting differences in preparatory-set. DeSouza and colleagues [17] further demonstrated an elevated BOLD response during the preparatory phase for anti-saccades compared to pro-saccades in the FEF and also in the right DLPFC and marginally in the intraparietal sulcus (IPS) bilaterally. The responses in these regions and also in the SEF, V1/V2 did not however differ between target-related and saccade-related activity for either the pro- or anti-saccade conditions. The elevated BOLD response for anti-saccade preparation in the frontal oculomotor regions is consistent with differences in the underlying saccade preparatory planning for the two types of eye movements [18].

The cortical oculomotor regions influence saccade planning via projections to the superior colliculus (SC) in the midbrain that in turn drives the brainstem saccade generator [19–21] although the nature of the signals conveyed by these projections is not fully understood [22]. The SC is a layered structure, in which the neurons in the superficial layers form a retinotopic visual representation of contralateral visual stimuli. The neurons in the intermediate layers also respond to visual stimuli and in addition discharge before a saccade of a specific direction and amplitude forming a ‘motor map’ of contralateral saccades [23–25]. Single cell recordings revealed decreased preparatory activity during the preparation for an anti-saccade compared to that observed during pro-saccade preparation [26]. The magnitudes of the stimulus-related and movement-related neuronal responses were reduced for the anti-saccade preparation period while activity of so-called fixation neurons showed enhanced activity [26]. Since the neural response in the SC associated with anti-saccades is attenuated it was suggested that additional inputs from other brain areas may be required for movement initiation following the target onset. This suggests that the SC might not process information about target selection, but might just be the structure executing the motor program required to generate a saccade. If this is right, then the SC could receive the information about response selection from cortical regions such as the FEF/SEF [26] and DLPFC [22]. The decreased neuronal activity in the SC contrasts with the increase in BOLD response in the cortical oculomotor regions for anti-saccades as described above.

We have recently applied fMRI to examine preparatory saccade-related activity in the human SC and observed that preparing a saccade produced an elevated BOLD response [27]. The increase in BOLD was observed in both the ipsilateral and contralateral SC. The BOLD signal was further increased during the saccade execution epochs and was significantly greater in the contralateral than ipsilateral SC. Having demonstrated preparatory activity for pro-saccades we here examine preparatory activity in the human SC and cortical oculomotor regions during preparation and execution of pro- and anti- saccades. Given the findings of reduced neural responses in primate SC for anti-saccades it is possible that the BOLD response will be attenuated. If, however, the fronto-parietal network mediates the processes involved in planning an anti-saccade, then the SC may have a more limited role in response preparation. We applied univariate and multivariate pattern analysis (MVPA) techniques to data acquired using a similar experimental paradigm to DeSouza and colleagues (2003) to examine the BOLD response in the human cortical oculomotor regions and in the SC. Activity associated with voluntary saccades has been successfully decoded in the intraparietal sulcus and precentral sulcus (regarded as the human homologue of the FEF) using MVPA but this technique has not been applied to decode activity the SC.

## Experiment 1: Preparatory-Set during the Preparation of Pro-Saccades and Anti-Saccades in the Cortex

The aim of this study was firstly to replicate the results found by DeSouza and colleagues [17] before applying the paradigm to the investigation of the human SC. The second aim was to extend those results by decoding the activity associated with pro-saccades and anti-saccades. To this end, we applied Multivariate Pattern Analysis (MVPA) (e.g., [28]) by training a Support Vector Machine (SVM) to decode the neural responses associated with two classes (anti-saccade, pro-saccade) during both the preparation phase and the execution phase of these eye movements.

## Materials and Methods

### Participants

Six healthy participants (3 females) took part in this experiment. All had normal or corrected to normal vision. They were screened for MRI contraindications according to standard procedures and written consent was obtained. The experimental procedure was in accord with the Declaration of Helsinki and was approved by the appropriate local ethics committee.

### Stimuli and task

Computer generated visual stimuli were projected by a LCD projector onto a rear-projection screen at the end of the scanner bore and were viewed via a mirror mounted on the headcoil, giving an image of 25° x 20° visual angle. The stimuli were created using a combination of MATLAB (The Mathwork, Inc.), ASF [29] and Psychtoolbox-3 [30,31].

**Fig 1:**
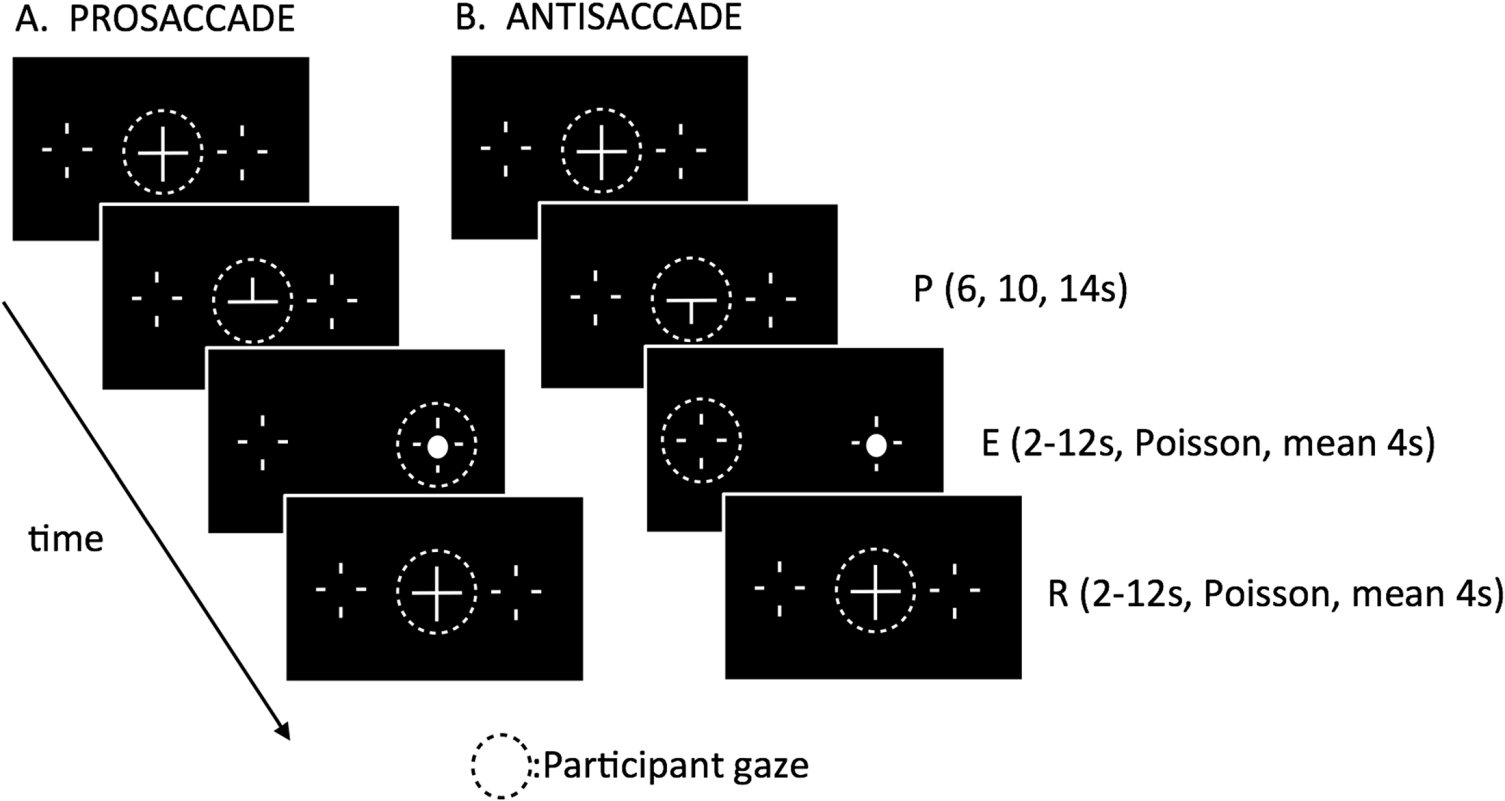
Diagram illustrating the two conditions used in the experiment. A: Pro-saccade trial. B Anti-saccade trial. The dotted circle indicates eye position at any given time and was not present on the screen. The panels show the successive events described in the text. Key: P = preparation, E = execution, R = return.

The stimuli are shown schematically in Fig 1. A white central fixation cross (0.5°) was presented on a black background, flanked by two continuously presented ‘landmarks’ placed at a distance of 5° left and right of fixation, that indicated the potential saccade goals. Each landmark comprised a white cross (diameter of 0.5°) with an inner empty black space (0.05°) representing the desired saccade landing position. The signal to execute a saccade was the onset of a small white circular target (0.05°) that appeared abruptly inside one of the saccade goals. There were two conditions as follows:

*Pro-saccade*—in this condition, at the start of each trial the lower leg of the central fixation cross disappeared indicating that the upcoming saccade should be made to the upcoming peripheral target onset. During the subsequent *saccade preparation* stage the participant was instructed to prepare a pro-saccade while keeping their gaze on the central fixation cross. During the preparation phase, the participant knew that a pro-saccade would be required but did not know its direction. To ensure a close replication of DeSouza et al (2003), the duration of the preparation phase was randomly chosen from 6s, 10s or 14s. At the end of this time, the fixation cross disappeared and, simultaneously, a target appeared inside one of the peripheral landmarks. The participant then performed a saccade to that location (*saccade execution* phase). Following the outward (centrifugal) saccade, gaze was held at the saccade target for a variable delay period, the duration of which was drawn from a Poisson probability distribution [32] with an average of 4 seconds, a minimum of 2 seconds and a maximum of 12 seconds. At the end of this time, the target disappeared and the central fixation cross reappeared. At this point, the participant had to perform a centripetal “return” saccade to the central fixation point. Gaze was then held on the central cross until the next trial commenced, after an inter-trial interval (ITI), again varying between 2s and 12s with a mean of 4s. The variation in the various delay intervals made it possible to separate the BOLD responses arising from the various events.

*Anti-saccade*—in this condition, the preparation phase was the same as in the ‘pro-saccade’ condition except that the upper arm of the central fixation cross disappeared, indicating that the upcoming saccade should be made in the direction opposite to the target onset. The participant then prepared to make an anti-saccade, again without knowledge of its direction. In the execution phase, the cross disappeared and a peripheral target appeared inside one of the landmarks, as in the pro-saccade condition. The participant then made an anti-saccade to the opposite, non-target location. All timings were as for the pro-saccade condition.

Each run contained 20 trials (10 pro-saccade and 10 anti-saccade). Half of the trials involved executing leftward saccades and half rightward saccades. The 20 trials were presented in random order within each run, with trials separated by a variable ITI, as described above. A black screen was presented for 15 seconds at the beginning and at the end of each run, allowing the BOLD signal to return to equilibrium. For each participant, 6 runs were conducted, using different randomizations.

### Data acquisition

Data were acquired using a 3T Siemens TIM Trio MR scanner with a 32 channel array head coil. Functional images were acquired with a *T*_2_*-weighted gradient-recalled echo-planar imaging (EPI) sequence (21 axial slices, interleaved ascending order with no gap between them, TR 1500 ms, TE 31 ms, flip angle 75°, resolution 3.0 mm isotropic, 64 x 64 matrix, FoV 192mm, bandwidth 752 Hz/Pixel, GRAPPA factor 2). The duration varied between scan runs according to the delay and ITI values selected from the probability distribution. The mean was 7 min 4s (281 volumes). Structural data were acquired using a *T*_*1*_-weighted 3D anatomical scan (MPRAGE, Siemens, TR 1830 ms, TE 5.56 ms, flip angle 11°, resolution 1x1x1 mm).

### Data analysis

Data were analysed using BrainVoyager QX 2.3 (Brain Innovation, The Netherlands). The first 2 volumes of each run were discarded to minimize T1 saturation changes. Three-dimensional motion correction with trilinear interpolation was performed using the first volume as a reference, followed by slice time correction. The data were then temporally high-pass filtered using a cut-off frequency of 3 cycle/run (~0.01 Hz). The first volume of the first run was used as a reference EPI scan for the alignment and for the coregistration. The preprocessed EPI scans were first aligned with the reference EPI scan, which was then coregistered with the anatomy. No spatial smoothing was performed on the functional data. The preprocessed data were analysed by running a general linear model (GLM) analysis with separate predictors for preparation (pro-saccade, anti-saccade) and execution (leftward pro-saccade, rightward pro-saccade, leftward anti-saccade, rightward anti-saccade). Each predictor consisted of an impulse (0.5s). For each participant, the six runs were combined and each event type was then modelled by convolving the predictor time course with a dual-gamma hemodynamic impulse response function (HRF) [33] and then scaling to unity.

Cortical activity was examined separately in each participant by defining three regions of interest (ROIs) corresponding to DLPFC, FEF and IPS, and averaging the blood-oxygen level dependent (BOLD) activity (beta values from the GLM) across all voxels within each ROI. The ROI was defined in the 3D space based on a *t*-map derived from the contrast between executed saccades (both pro-saccade and anti-saccade trials, left and right sides pooled) and baseline activity. The resulting patches of activity at the known anatomical location of each region were identified for each subject individually after suitable thresholding of the *t*-map (FDR(q) < 0.001) and taken as the ROI. Defining ROIs based on saccade execution permits measurement of saccade preparation activity within an independently defined ROI. It of course produces a potential bias towards greater activity for execution than preparation, but this is unimportant for our purpose. To quantify the effect of preparing a saccade, the beta estimates were averaged across left and right ROIs. The resulting parameter estimates were examined across participants and across ROIs by submitting them to a two-way Analysis of Variance (ANOVA) for repeated measures, with ‘saccade type’ (prosaccade, antisaccade) and ‘saccade phase’ (preparation, execution) as within-subject factors. The data were further submitted to a *post hoc* testing where the contrasts of interest were examined by means of paired t-test.

The multivariate analysis was based on exemplars that consisted of beta values from the GLM analysis conducted as above. We first combined, individually for each subject, the voxels from the three left-hemisphere ROIs with those from the corresponding right ROIs to yield a set of three ROIs per participant. Then we ran the univariate analysis using five regressors: (preparation and execution for each of pro-saccades and anti-saccades plus execution of return saccades). The saccade directions (leftward, rightward) were collapsed as the ROIs were concatenated between hemispheres. The beta estimates resulting from this univariate analysis were then submitted to multi-voxel pattern analysis (MVPA).

We ran a MVPA to test the hypothesis that pro-saccades and anti-saccades could be distinguished (decoded), during both the preparation phase and the execution phase. We therefore ran the MVPA separately on the beta values from the preparation phase and on those from the execution phase. Separate analyses were conducted for each ROI, based on all voxels in the ROI. A limitation of this ROI-based approach is that small cortical areas (or SC in the Experiment 2) may not contain enough functional voxels to run a successful MVPA analysis. To ameliorate this problem, data were concatenated across participants prior to MVPA analysis [34,35]. The beta values were normalised to remove any overall difference between the classes and then used as trial response values (exemplars). Within each participant, all beta values were first normalised to 1.0 to remove magnitude differences between participants. The responses for each class were then normalised to 1.0 within each participant to remove magnitude differences between classes. Decoding performance was examined for each ROI as a function of the number of features included by progressively including more voxels, selected randomly, and repeating the analysis. For each sample size, the analysis was repeated 20 times with a different random selection of voxels and the resulting decoding performances were averaged.

For each MVPA, a subset of observations was used to train the classifier, which was a Support Vector Machine (SVM) with a linear kernel. The SVM was trained to identify the optimal separating boundary (hyperplane) between the two Classes (i.e., pro-saccade, anti-saccade). A ‘leave-one-out’ method was used. Of the 6 runs, 5 were used for training and the 6^th^ was used for testing. This was repeated 6 times, leaving out each run in turn, and the 6 performances were averaged. Finally, for each ROI, the hypothesis that the classification accuracy was different from chance level was tested by comparing it against the test accuracy on the same dataset after having randomly permuted (shuffled) the labels, which should produce chance-level accuracies with a similar variance to the main analysis. 1000 such analyses were performed with different random permutations, employing the same leave-one-out method, giving 1000 performance estimates per permutation. The 95^th^ percentile of the distribution of permuted performance results was taken as a critical value for regarding un-permuted performance values as significantly above chance. GLM analysis was performed with BrainVoyager and all analyses beyond GLM (merging the ROIs, voxel selection, SVM classification) were performed with MATLAB (The Mathwork, USA) using the LIBSVM library for support vector machines [36].

### Eye movement recording

In order to check that saccades were initiated at the correct time and in the correct direction in relation to the cue and target, eye position was recorded using an infrared video camera (NordicNeuroLab, Norway) positioned close to the eye inside the scanner. Pupil position was continuously sampled with a frequency of 60Hz using software (Arrington, Inc. USA) that located and tracked the pupil. The camera image was continuously monitored on-line to ensure the participant was following the task instructions. Where possible, eye position was analysed off-line to determine the error rate for saccade direction but due to loss of tracking for some participants, eye position could not be analysed in detail in all cases. During the preparation phase, errors were defined in terms of eye movements made in association to the cue when fixation should be maintained. During the pro-saccade and anti-saccade execution phases, errors were defined in terms of saccades not generated during the execution interval and saccades made in the incorrect direction.

## Results

### Eye trace analysis

The analysis of the eye-tracking records showed that participants produced very few errors. Data for one participant could not be examined (due to noise in the record) but the analysis for the remaining 5 participants showed that they did not generate saccades during the preparation phase. The overall direction error rates in the execution phase was ~1%, for both pro-saccades and anti-saccades. Given the low amount of errors all the trials were included in the fMRI analysis.

### ROI definition: Activation of DLPFC, FEF and IPS during saccade execution

**Fig 2:**
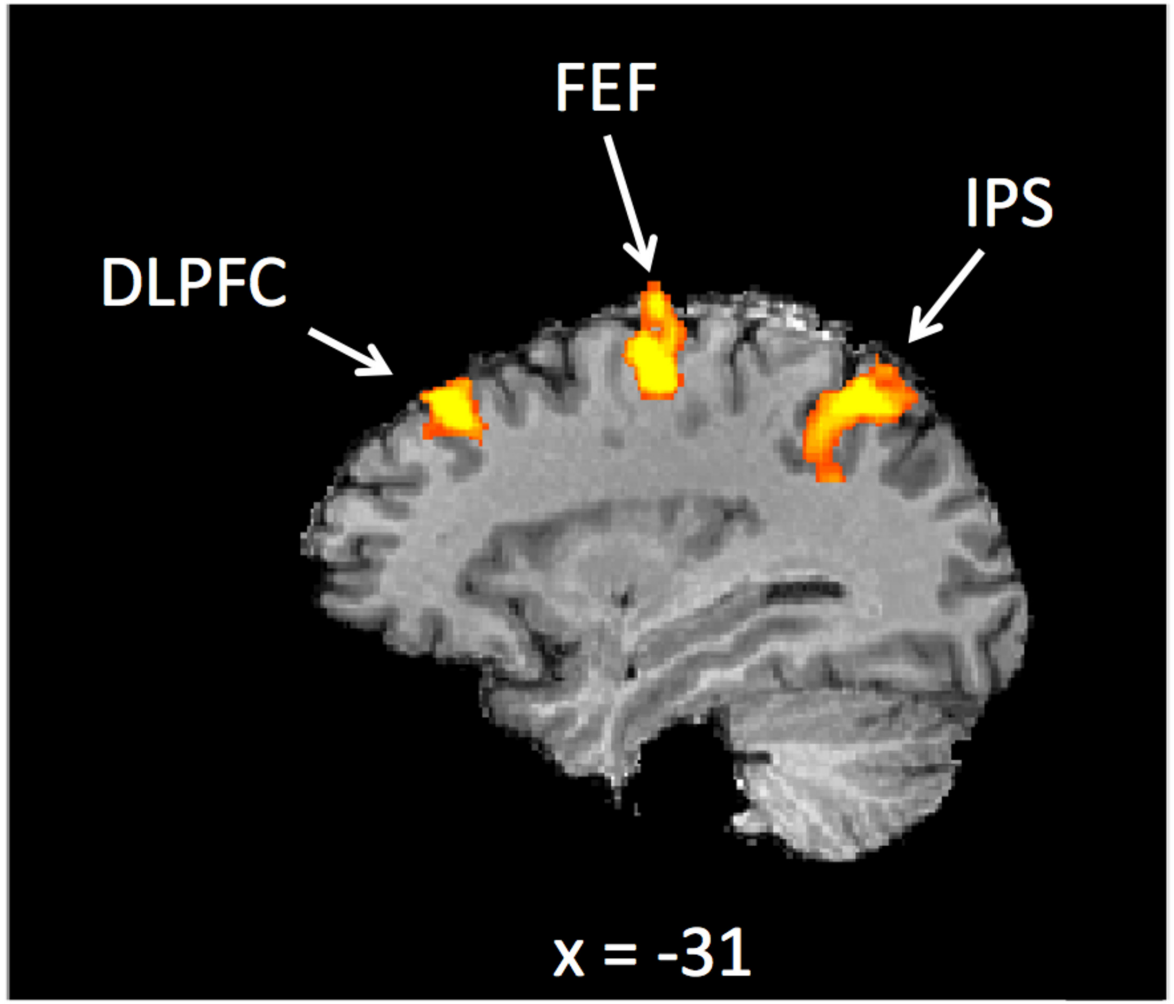
Locations of the DLPFC, FEF and IPS in one representative subject. Executing a saccade (pro-saccade, anti-saccade) was used as an event to functionally identify the six ROIs (DLPFC, FEF, IPS in each of the two hemispheres). These regions are consistent with those localized by DeSouza et al (2003) and the Talairach coordinates are shown in Table 1.

Fig 2 shows a statistical parametric map for saccade execution in pro-saccade and anti-saccade trials (leftward and rightward saccades pooled), on which the ROI definition was based, for one representative participant. The threshold was set at p < 0.0001 (unc.) as in [17]. The analysis of the voxel-wise statistical map revealed significant bilateral activation in the DLPFC, FEF and IPS for all six participants. The ROI locations averaged across all analysed participants (n = 6), expressed in Talairach coordinates, are shown in Table 1.

**Table 1:** Talairach coordinates (μ±σ, x, y, z, volume) averaged across six participants included in the analysis.

	X (μ±σ)	Y (μ±σ)	Z (μ±σ)	Volume (mm^3^)
DLPFC				
LH	27±3	32±4	31±3	1243
RH	-28±2	29±3	33±3	540
FEF				
LH	-29±6	-11±3	48±5	2995
RH	31±8	-9±4	46±4	3720
IPS				
LH	-25±5	-57±5	42±5	2254
RH	23±4	-55±4	41±4	1858

### Cortical activation during the execution of ipsilateral and contralateral saccadic eye movements

**Fig 3:**
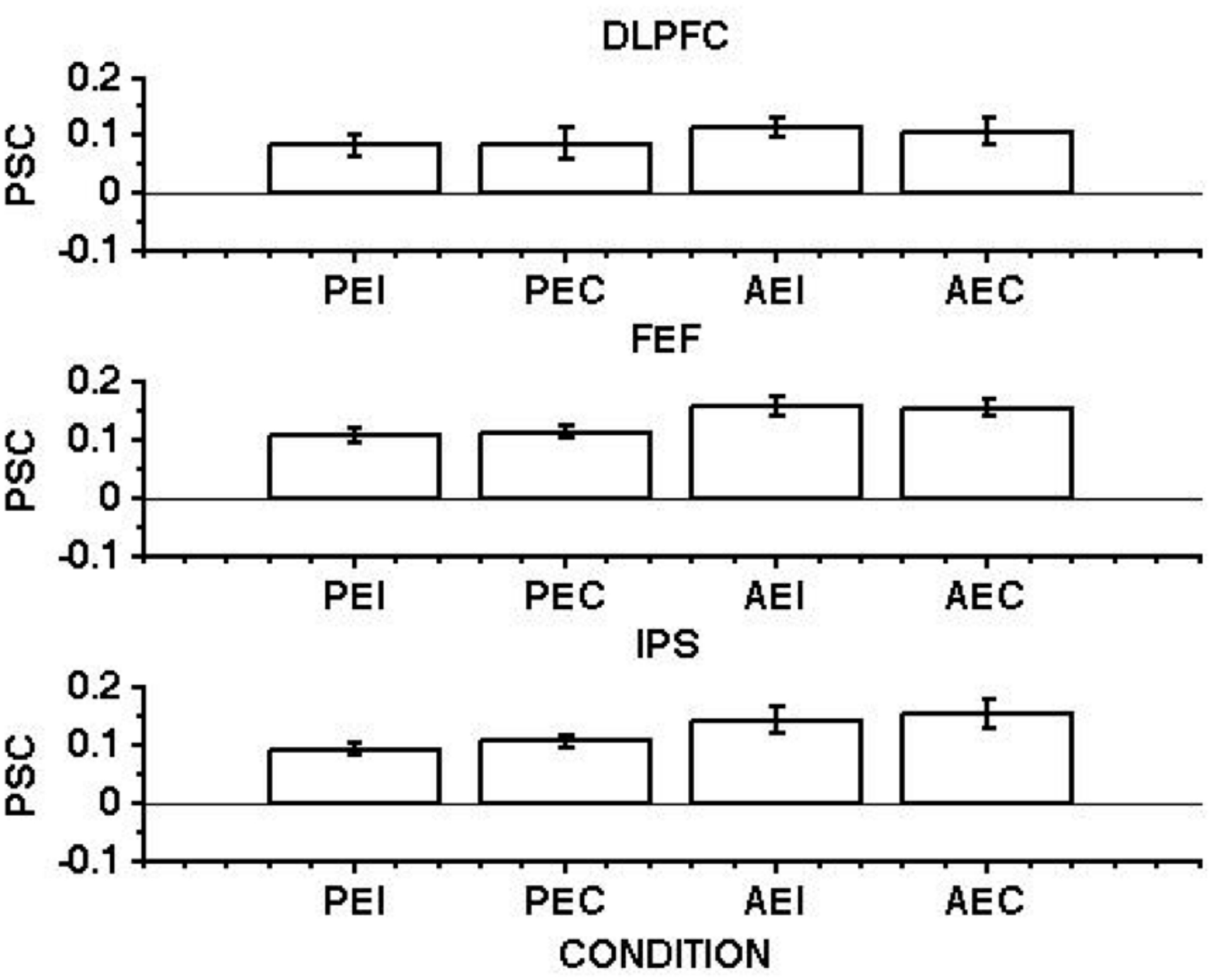
BOLD responses averaged across 12 hemispheres from 6 subjects for ipsilateral and contralateral execution of pro-saccades and anti-saccades, shown for the DLPFC (upper row), FEF (middle row) and IPS (bottom row). Starting from the left, the first two bars show the activity time-locked to the onset of the cue to execute an ispilateral (PEI) and a contralateral pro-saccade (PEC). The third and fourth bars show the activity time-locked to the cue to execute an ispilateral (AEI) and a contralateral anti-saccade (AEC).

We first measured the presence of a contralateral bias in BOLD response relating to response direction. Specifically, we checked whether generating a contralateral saccade produced a larger response than did generating an ipsilateral saccade. Leftward and rightward saccades were modelled separately and then the beta estimates produced by all the saccadic events organised in terms of ipsilateral and contralateral saccades, i.e. rightward saccades were considered ipsilateral when referred to the ROIs in the right hemisphere, and contralateral when referred to the ROIs in the left hemisphere. Responses relating to ipsilateral and contralateral saccades were then pooled between the two hemispheres. As a result of this process, the left and the right ROIs were concatenated. This analysis provided us with a matrix of beta estimates referred to as ipsilateral and contralateral saccades, separated for three ROIs. Fig 3 shows the saccade response magnitude related to the execution of ipsilateral and contralateral pro-saccades and anti-saccades.

Generating a pro-saccade toward either the ipsilateral or contralateral target generated hemodynamic responses that were not significantly different, in the DLPFC (t_(11)_ = -0.1244, n.s.), FEF’s (t_(11)_ = -0.6794, n.s.) and in the IPS (t_(11)_ = -2.0119, n.s.). The same pattern was observed for anti-saccades, in the DLPFC (t_(11)_ = 0.4715, n.s.), FEF’s (t_(11)_ = −0.1884, n.s.) and in the IPS (t_(11)_ = -0.5916, n.s.). Table 2 reports means and standard errors of normalised percentage signal change for each saccade event type.

**Table 2:** Mean and standard error of normalised signal change for each saccade event type.

	PROSACCADE		ANTISACCADE	
	EXECUTION		EXECUTION	
	IPSILATERAL (μ±σ)	CONTRALATERAL (μ±σ)	IPSILATERAL (μ±σ)	CONTRALATERAL (μ±σ)
DLPFC	0.0827±0.0184	0.0858±0.0268	0.1144±0.0162	0.1067±0.0224
FEF	0.1049±0.0121	0.1111±0.0104	0.1563±0.0161	0.1528±0.0146
IPS	0.0900±0.0104	0.1045±0.0111	0.1410±0.0229	0.1512±0.0258

Since there was no difference in terms of response magnitude between ipsilateral and contralateral saccades, these were collapsed together for subsequent analysis.

### Cortical activation during the preparation and execution of pro-saccades and anti-saccades

**Fig 4:**
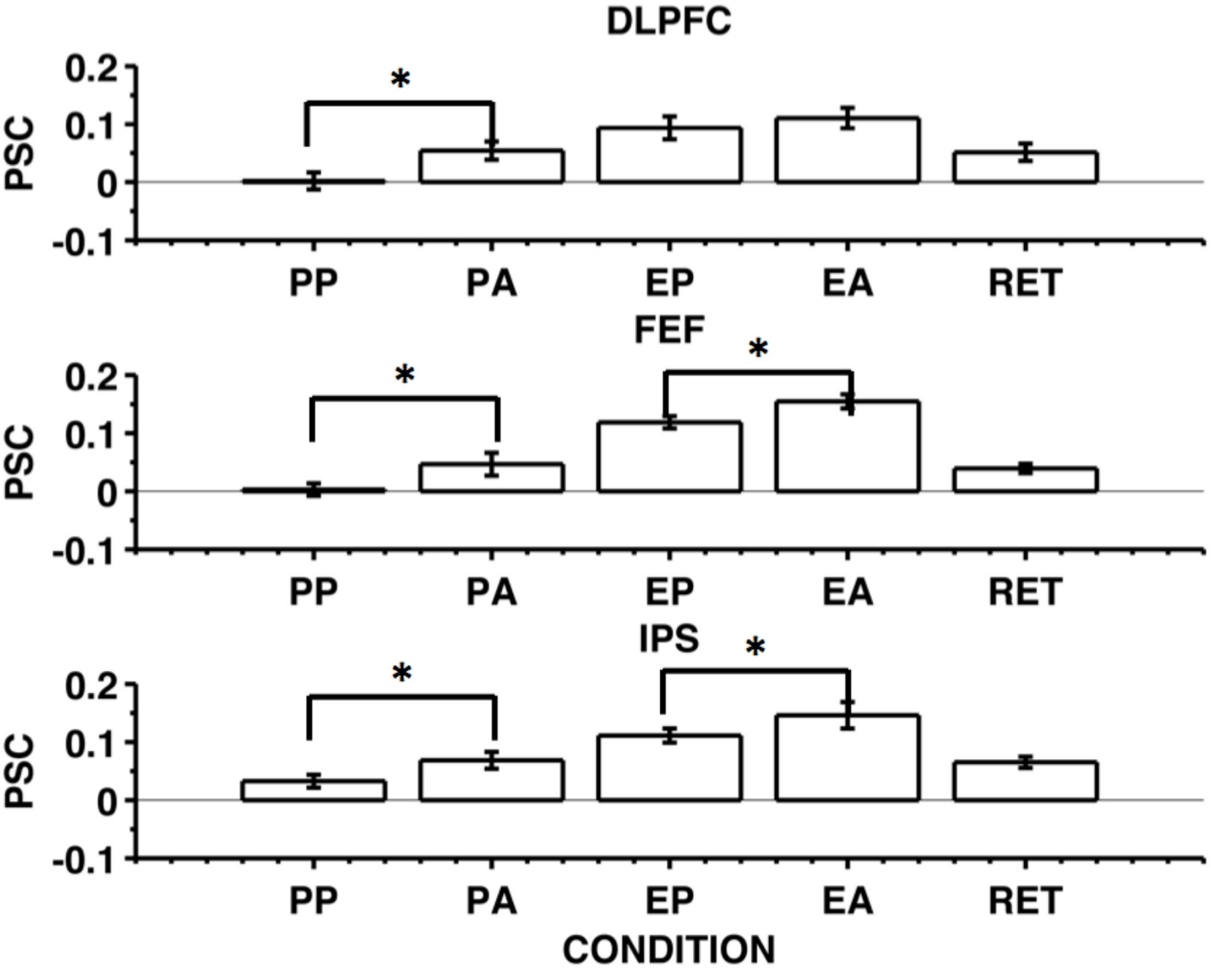
BOLD responses averaged across 12 hemispheres from 6 subjects for preparation and execution of pro-saccades and anti-saccades, shown for the DLPFC (upper row), FEF (middle row) and IPS (bottom row). Starting from the left, the first two bars shows the activity time-locked to the onset of the cue to prepare a pro-saccade (PP) and an antisaccade (PA). The third and fourth bars show the activity time-locked to the cue to execute a pro-saccade (EP) and an anti-saccade (EA). The last bar on the right shows the activity time-locked to the cue to perform a return saccade from the peripheral landmark to the central fixation cross (RET).

Our initial objective was to replicate the findings of DeSouza (2003), prior to performing MVPA. Specifically, we aimed to measure whether anti-saccades produced greater BOLD responses than pro-saccades, during both the preparation and execution of saccades. Fig 4 shows saccade response magnitude for pro-saccades, anti-saccades and return saccades. The responses related to pro-saccades and anti-saccades are shown separately for the preparation phase (PP, PA) and the execution phase (EP, EA). This distinction is not possible for the centripetal return saccades (RET) as these saccades are always directed to the central fixation point.

The hemodynamic activity increased with the type of saccade produced (pro-saccade, anti-saccade) with greater activity for anti-saccades in the DLPFC [F_(1,11)_ = 15.4, p = 0.002], in the FEF [F_(1,11)_ = 26.33, p < 0.001] and in the IPS [F_(1,11)_ = 13.15, p = 0.004]. The hemodynamic activity increased with the phase of a saccade (preparation, execution) in the DLPFC [F_(1,11)_ = 38.57, p < 0.001], in the FEF [F_(1,11)_ = 66.89, p < 0.001] and in the IPS [F_(1,11)_ = 33.05, p < 0.001].

A possible explanation of the greater activity for anti-saccade execution might be that after execution, the cue for the return saccade is further away from fixation than for pro-saccades, so detecting it may require greater attention. This might elevate BOLD during the interval between the outward and return saccades and some of this increase might be captured by the model used for the outward saccade, even though it arises later than the saccade itself. However, because the delay has a minimum of 2 sec, participants need not attend immediately and any such contamination is likely to be minimal. In addition, a similar difference between pro- and anti-saccades was seen for saccade preparation, which is too temporally remote to be affected in this way.

Further exploration by means of *post hoc* contrasts revealed that the preparatory phase of pro-saccades produced a BOLD response indistinguishable from the baseline activity, in either the DLPFC (t_(11)_ = 0.13, ns) or the FEF’s (t_(11)_ = 0.26, ns). However, preparing a pro-saccade produced an increase in the IPS (t_(11)_ = 2.91, p = 0.001). In contrast, during the preparatory phase of anti-saccades a significant increase in the response was observed in all three ROIs (DLPFC: t_(11)_ = 3.46, p = 0.005; FEF: t_(11)_ = 2.37, p = 0.03; IPS: t_(11)_ = 4.69, p < 0.001). The comparison between the preparatory phases of pro-saccades and anti-saccades revealed that anti-saccades produced a larger BOLD response than pro-saccades in the DLPFC (t_(11)_ = -4.06, p = 0.002), the FEF (t_(11)_ = -3.04, p = 0.01) and the IPS (t_(11)_ = -3.23, p = 0.008).

Executing a saccade produced a significant increase of the BOLD signal in all of the ROIs. Executing a pro-saccade increased the BOLD signal in the DLPFC (t_(11)_ = 4.39, p = 0.001), the FEF (t_(11)_ = 10.47, p < 0.001) and the IPS (t_(11)_ = 9.6, p < 0.001). Executing an anti-saccade produced a similar effect, with increased activity observed in the DLPFC (t_(11)_ = 6.23, p < 0.001), the FEF (t_(11)_ = 12.71, p < 0.001) and the IPS (t_(11)_ = 6.40, p < 0.001). Executing a pro-saccade produced a larger response than in the preparation phase in all three ROIs. It should be noted, however, that this may reflect a bias resulting from the ROIs being defined using the response for saccade execution that produces a potential overestimation of execution activity compared to preparation activity (which is unbiased). Furthermore, activity in the execution phase includes the preparatory activity and it is likely therefore that although it appears less than “execution” activity in Fig 4 it may be at least comparable in magnitude to execution-related activity as was also observed by DeSouza et al (2003).

A comparison of the two conditions showed that executing anti-saccades produced a significantly larger neural response than pro-saccades in the FEF (t_(11)_ = -3.10, p = 0.01) and in the IPS (t_(11)_ = -2.54, p = 0.02), but not in the DLPFC (t_(11)_ = -1.28, p = ns). Measurable responses were also observed during centripetal (return) saccades made back to the central fixation point in all the cortical regions examined here (DLPRC: t_(11)_ = 3.43, p = 0.006; FEF: t_(11)_ = 4.87, p < 0.001; IPS: t_(11)_ = 6.75, p < 0.001) although this was smaller than for the execution of outward pro- (DLPRC: t_(11)_ = 2.21, p = 0.049; FEF: t_(11)_ = 4.83, p < 0.001; IPS: t_(11)_ = 2.37, p < 0.036) and anti- saccades (DLPRC: t_(11)_ = 8.18, p < 0.001; FEF: t_(11)_ = 6.41, p < 0.001; IPS: t_(11)_ = 4.26, p = 0.001). Table 3 reports means and standard errors of normalised percentage signal change for each saccade event type.

**Table 3:** Mean and standard error of normalised signal change for each saccade event type.

	PREPARATION		EXECUTION		RETURN
	PROSACCADE (μ±σ)	ANTISACCADE (μ±σ)	PROSACCADE (μ±σ)	ANTISACCADE (μ±σ)	(μ±σ)
DLPFC	0.0019±0.0147	0.0541±0.0156	0.0843±0.0192	0.1106±0.0177	0.0513±0.0150
FEF	0.0028±0.0106	0.0465±0.0196	0.1080±0.0103	0.1546±0.0122	0.0391±0.0080
IPS	0.0325±0.0112	0.0685±0.0146	0.0972±0.0101	0.1461±0.0228	0.0652±0.0097

### Decoding pro-saccades and anti-saccades in the DLPFC, FEF and IPS

**Fig 5:**
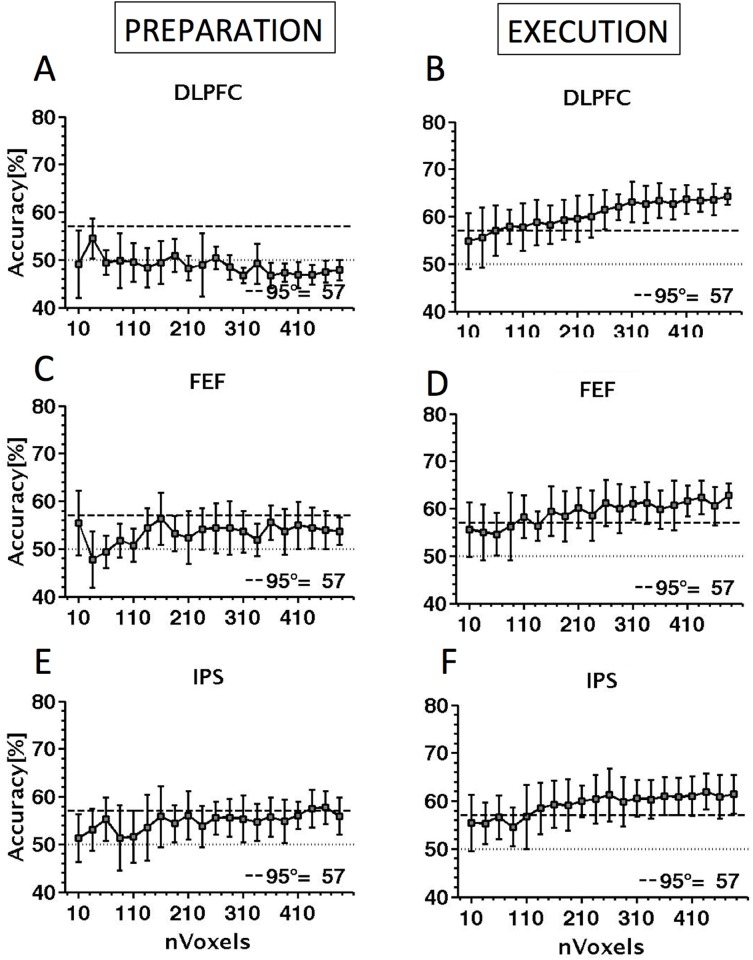
Decoding performance for saccade type (pro-saccade vs. anti-saccade) for the three cortical regions examined. In each plot, performance is shown as a function of voxel sample size (number of exemplars) included in the MVPA analysis. Decoding performance is shown separately for the preparation phase (A, C, E) and the execution phase (B, D, F), for the DLPFC (A, B), the FEF (C, D) and the IPS (E, F). Each point is the mean performance from 20 random voxel selections of the size shown on the abscissa. Also shown are the chance decoding performance (dotted line) and the performance level that is significantly above chance at p<0.05 derived by permutation testing (dashed line).

Fig 5 shows the ability of the SVM to decode (predict) which class a given stimulus belonged to as a function of the number of voxels (exemplars) included in the analysis. Also shown in the figure is the chance level accuracy (50%, dotted line), together with the 95^th^ percentile of the distribution of accuracies based on permutation testing in the relevant cortical area.

All three regions studied reliably supported decoding of saccade type during the execution of a saccade. In each case, the accuracy of the classifier increases monotonically with the sample size, peaking at 64.25% in the DLPFC, 62.75% in the FEF and 61.95% in the IPS. Decoding during the preparation phase was more elusive: in DLPFC, performance was at chance for all sample sizes, suggesting that it is not sensitive to the difference between prosaccades and antisaccades during planning. In FEF, performance was marginally above chance but shows no dependence on sample size and never reached statistical significance. The only suggestive evidence for decoding is in IPS, where performance reaches marginal significance for the largest voxel samples and also shows a systematic increase with sample size, an important hallmark of genuine decoding performance.

**Fig 6:**
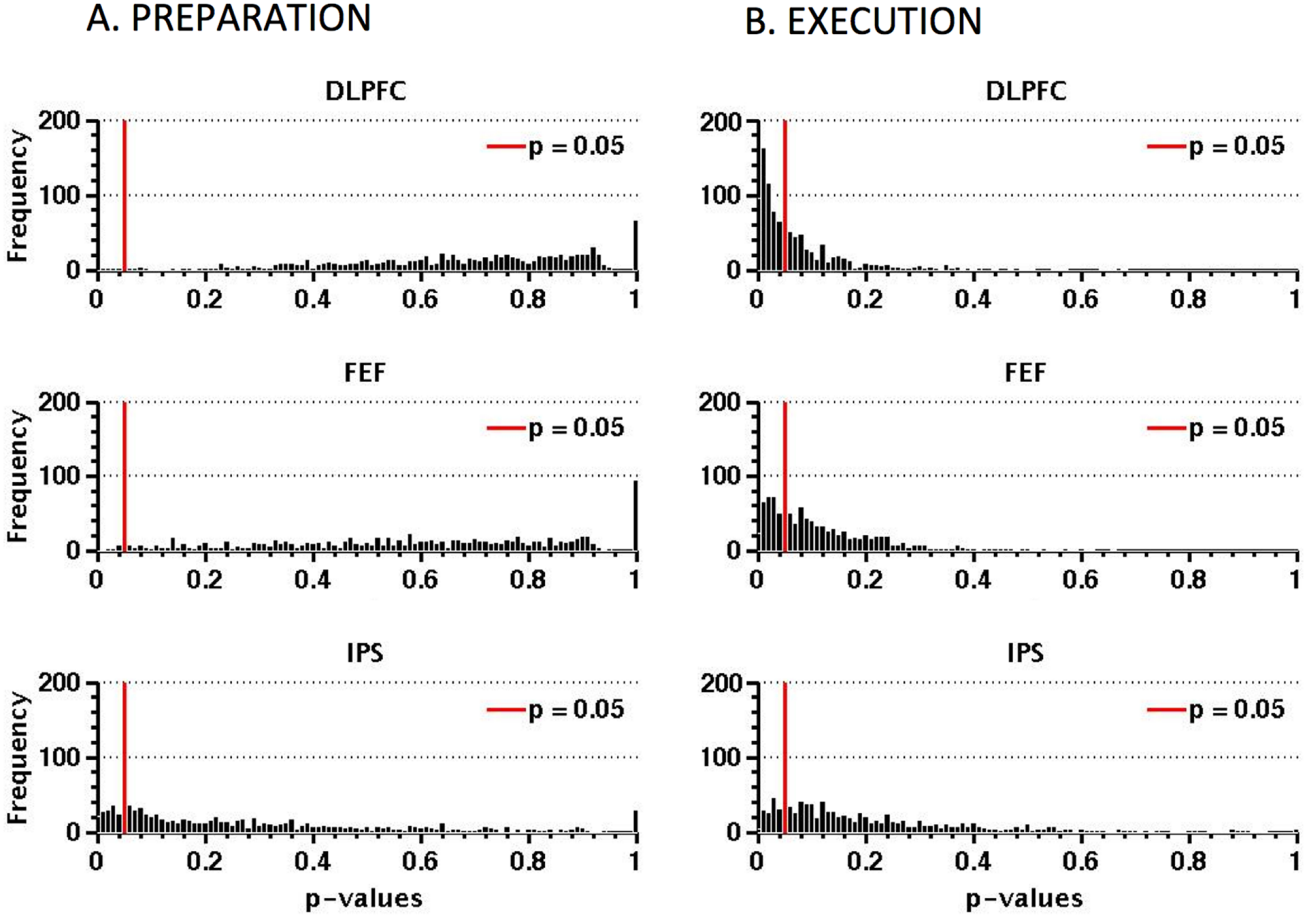
Results from an analysis in which decoding performance during the preparation phase (A) and the execution phase (B) (based on 500 voxels) was compared with the performance in the same sample when the labels (pro-saccade, anti-saccade) associated with the exemplars were randomly permuted. 1000 t-tests were performed with different voxel samples and label permutations. The binned frequency of p-values associated with the t-tests is shown as a function of p-value. Clustering at low values indicates strong sensitivity to saccade type.

Another way to evaluate the data is to take only the largest voxel sample (end point of plots in Fig 5) and consider the distribution of probability values obtained when decoding performance for samples of this size is compared by *t*-test with performance in permutation tests. Strong decoding performance should be reflected in the *p* values clustering at low (significant) values (shown in Fig 6A and 6B). For each cortical area, 500 voxels were randomly selected. Decoding performance was evaluated for this sample with correct and randomly permuted labels. This was repeated 1000 times with a different random sample of 500 voxels and a different permutation each time, yielding a total of 1000 *p* values. For the preparatory phase (Fig 6A) the DLPFC has few p-values below the significant threshold of 0.05 (9), followed by the FEF (15) while the IPS has more (151) which is suggestive of decoding. For the execution phase (Fig 6B) the clustering at low *p* values is evident in all three areas during the execution phase with the DLPFC has the largest number of p-values below the threshold of 0.05 (541 values), followed by the FEF (307) and the IPS (172). The results considered in this way are in line with the raw decoding performances shown in Fig 5.

## Discussion: Cortical Activity

Experiment 1 successfully replicated and extended the results reported by DeSouza and colleagues with the BOLD response associated with the preparation of anti-saccades being greater than for pro-saccades in the DLPFC and FEF’s. In addition in our study the IPS also showed a significant response elevation during the preparation of anti-saccades. The involvement of the IPS is consistent with Curtis and Connolly [16] who reported greater IPS activity for anti-saccade than pro-saccade preparation although in their study this was observed only when upcoming saccade direction was known. Here the DLPFC, FEF and IPS all show greater responses associated with the preparation for an anti-saccade when direction is unknown suggestive of preparatory-set [37]. By contrast, the DLPFC and the FEF were largely unresponsive during the preparation of pro-saccades. Executing pro-saccades and anti-saccades produced a significant increase in the BOLD response in DLPFC, the FEF and the IPS. However, executing an anti-saccade produced a larger BOLD response, compared to that observed for pro-saccades in the FEF and in the IPS, but not in the DLPFC. The cortical activity associated with the centrepetal return saccades was significantly lower than that for outward pro- and anti- saccades in all three ROI’s. This differs from the results of Krebs et al. [38] who did not observe a reduced cortical BOLD signal for return saccades (although it was reduced in the colliculus c.f. [27]).

The MVPA classifier analysis was not able to reliably decode pro- and anti-saccades during the preparatory period in the DLPFC or FEF, but there was some evidence of decoding in the IPS. By contrast, for saccade execution pro- and anti-saccades could be reliably decoded in all the three cortical regions of interest. Specifically, the SVM successfully predicted pro-saccade and anti-saccade 62.7% in the FEF and 61.9% in the IPS, showing that these regions encode the motor program underlying the execution of pro-saccades and anti-saccades. A similar result was observed in the DLPFC, where the classifier decoded the two types of saccades 64.25% of the time. Our findings are at least partially consistent with Chan et al. (2015) [39] who showed that the SVM predicted the saccade type in several cortical regions, among these the FEF and IPS. However, the highest accuracy observed by Chan and colleagues was 57.3% in the FEF and 58.8% in the IPS. Although these values are above 50%, commonly taken as chance level this does not take into account the null distribution of accuracies. We think that a more reliable way to analyse the data obtained with MVPA is to calculate the null distribution and then compare the accuracy in decoding pro- from anti-saccade with the 95^th^ percentile of the null distribution [35].

## Experiment 2: Preparatory-Set during the Preparation of Pro-Saccade and Anti-Saccades in the Superior Colliculus

Having successfully demonstrated preparatory activity in the FEF, DLPFC and IPS we then investigated preparatory activity in the SC associated with pro-saccades and anti-saccades using the same paradigm. The aims of this second experiment were twofold. First, we aimed to replicate our own findings of saccade-related preparatory activity in the SC [27] and secondly we want to extend this to investigate higher-level cognitive functions involved in planning an anti-saccade in the SC. Patterns of activity in the SC may be expected to differentiate between pro and anti saccades due to differences in pre-response activity in populations of saccade-related neurons in caudal regions and fixation neurons in the rostral region. It has been proposed that the pre-frontal cortex inhibits the visual-grasp reflex in the anti-saccade task by enhancing activity in fixation neurons and inhibitory interneurons in the ipsilateral SC {Johnston, 2006 #2174. An alternative model is that the DLPFC sends an excitatory signal to the caudal saccade-related neurons that could be attenuated in the anti-saccade task {Johnston, 2014 #2404. Although these models differ in terms of how the cortical input to the SC can serve to control voluntary saccades both predict differential patterns of activity associated with pro and anti-saccades that may be revealed by MVPA.

## Materials and Methods

### Participants

Nine healthy participants (6 females) took part in this experiment. Of these, five had participated in Experiment 1. All had normal or corrected to normal vision.

### Stimuli and task

Visual stimuli were generated as described for Experiment 1 (see Stimuli and task).

### Data acquisition and analysis

Data analysis was the same as for Experiment 1. The only difference concerns how the hemodynamic response was modelled. In Experiment 2, we optimised the signal estimation within the SC by convolving each predictor time course with a HRF with an early peak (4.5 seconds), which has been demonstrated to be better suited to modelling hemodynamic activity in the SC {Wall, 2009 #375}.

Data acquisition was slightly different with respect to Experiment 1, due to the size and location of the SC. The structural data were acquired with the same sequence described in Experiment 1. Functional images were acquired with a *T*_2_*-weighted gradient-recalled echo-planar imaging (EPI) sequence (16 axial slices, TR 1500, TE 41 ms, flip angle 75°, resolution 2.0 mm isotropic, 96 x 96 matrix, FoV 192mm, bandwidth 752 Hz/Pixel, GRAPPA factor 2). As in Experiment 1, the duration varied between scan runs according to the delay and ITI values selected. The mean was 7 min 4s (281 volumes).

In Experiment 2, we defined two ROIs, separately for each participant, corresponding to left and right SC by selecting the voxels overlapping the anatomical location of each colliculus. In the current experiment the ROIs were defined anatomically instead of functionally. For the SC it is possible to identify clear anatomical landmarks to define it anatomically (which was not possible in the case of the cortical regions examined in Experiment 1). The border where the SC meets the cerebrospinal fluid (CSF) defines the lateral and caudal portion, the lower portion is defined by the connection between the SC and the inferior colliculus, the boundary with the tectum defines its upper extent and the boundary with the periacqueductal grey defines the rostral extent.

Having identified the ROIs, the mean BOLD response magnitudes (β values) corresponding to each condition were calculated by averaging across all voxels in the ROI. In order to remove any between subjects bias, the resulting parameter estimates were normalized as described in Experiment 1, and then tested for significant activity across participants by *t*-tests. The multivariate analysis was conducted as in Experiment 1.

### Eye movement recording

Eye movements were again monitored on-line to ensure participants were following the task instructions as for Experiment 1. A further qualitative analysis on the eye traces was performed as explained in Experiment 1.

## Results

### Eye traces analysis

The eye position analysis revealed that participants produced very few errors. The eye records for two participants were too noisy for analysis but the records for the remaining 7 participants showed that they did not generate saccades during the preparation phase. The overall direction error rates in the execution phase was ~1%, for both pro-saccades and anti-saccades, which is consistent what we observed in Experiment 1. Given the low amount of errors produced by the participants, all the trials were taken in account for the analysis.

### ROI definition

**Fig 7:**
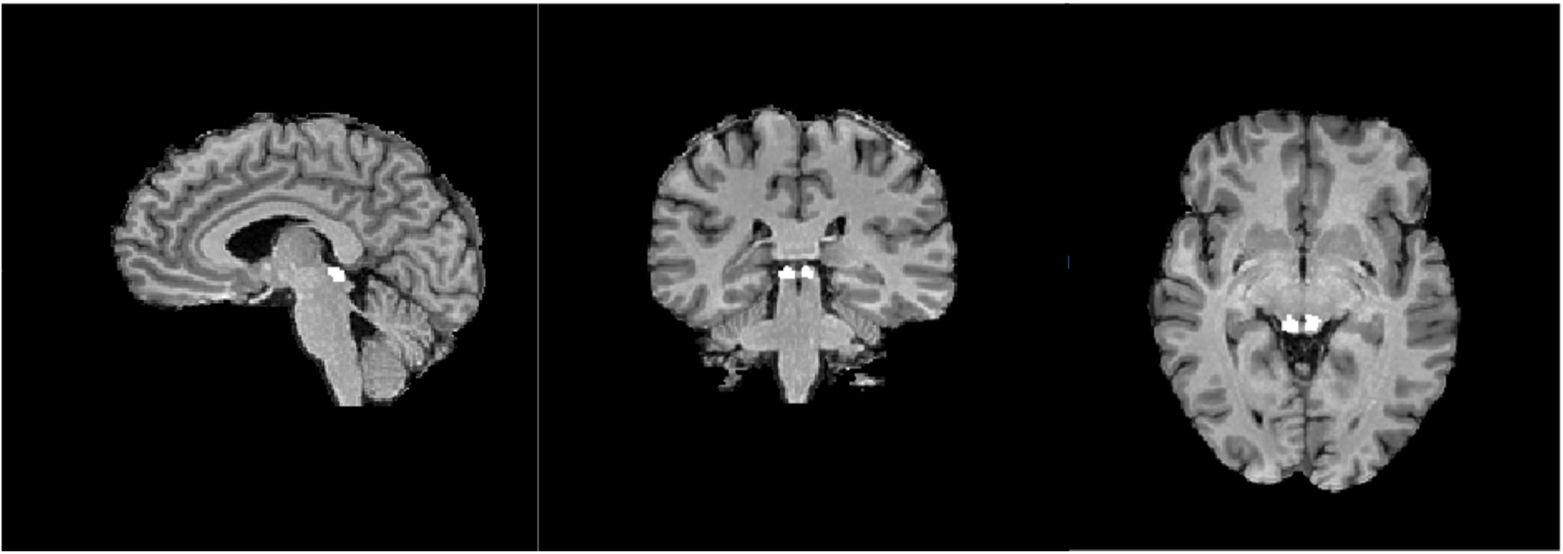
Locations of the anatomically defined left and right SC ROIs in one representative subject.

Fig 7 shows the location of our anatomically defined SC for one participant. The mean ROI locations across participants, expressed in Talairach coordinates, are shown in Table 4.

**Table 4:** Mean and standard error of normalised signal change for each saccade event type in the SC.

	X	Y	Z	Volume (mm^3^)
SC LH	-4	-28	-3	209
SC RH	6	-29	-3	250

### Collicular activation during the execution of ipsilateral and contralateral saccades

**Fig 8:**
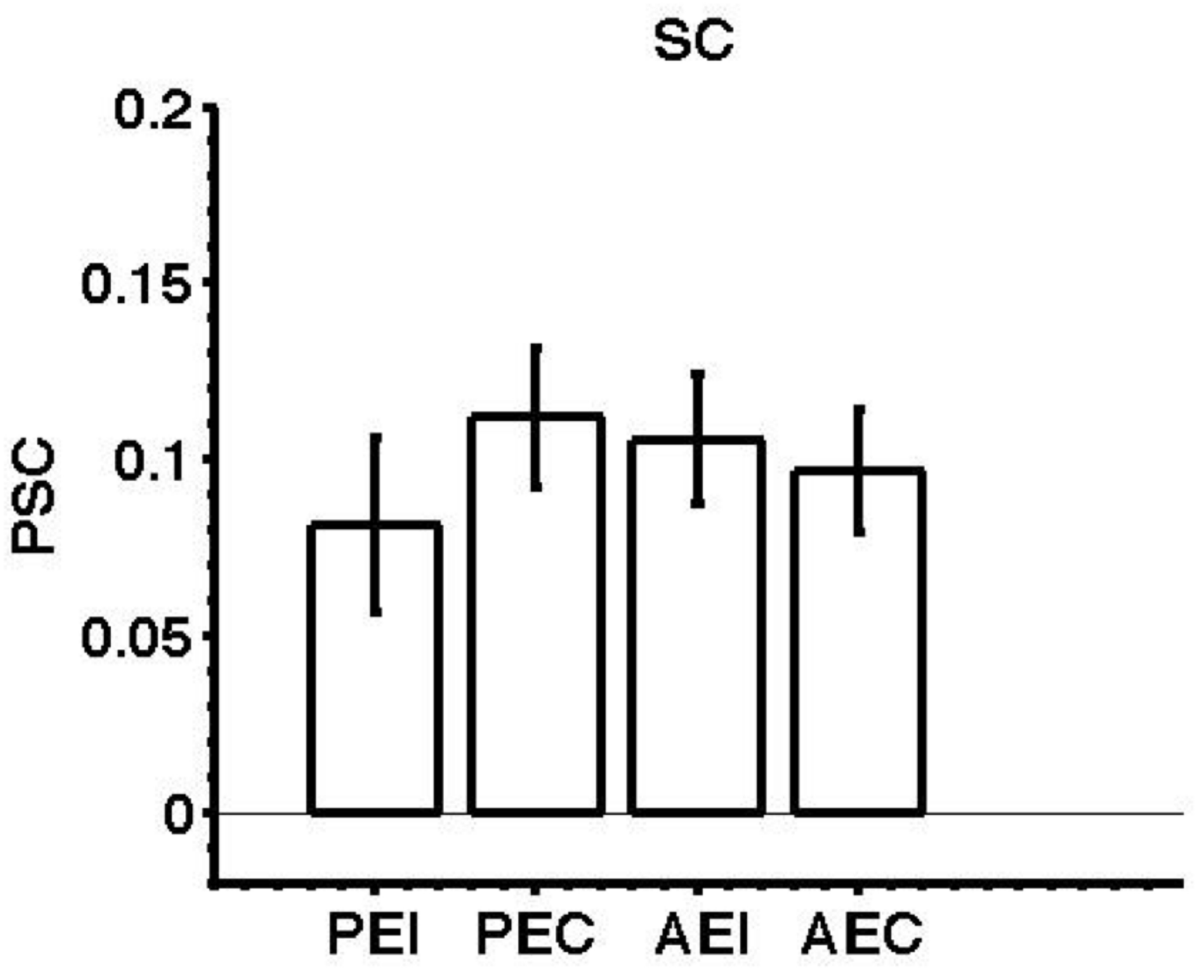
BOLD responses averaged across 18 hemispheres from 9 subjects for ipsilateral and contralateral execution of pro-saccades and anti-saccades, in the SC. Key as for Fig 3.

In Experiment 2 we first measured the contralateral preference in the human SC, as described for the cortical regions above. Activity associated with leftward and rightward saccades was modelled separately, the beta estimates associated with all the saccadic events separated in relation to ipsilateral and contralateral saccades. Responses for ispilateral saccades from the left SC were concatenated with those for ipsilateral saccades in the right SC and the same process was performed for activity associated with contralateral saccades. Fig 8 shows the saccade response magnitude related to the execution of ipsilateral and contralateral pro-saccades and anti-saccades.

Generating a pro-saccade toward either the ipsilateral or the contralateral target generated response amplitudes that were not significantly different (t_(17)_ = -1.2627, n.s) and the same was the case for anti-saccades (t_(17)_ = 0.7711, n.s.). Table 5 reports means and standard errors of normalised percentage signal change for each saccade event type. Since there was no difference in terms of response magnitude between ipsilateral and contralateral saccades activity was collapsed across saccade direction.

**Table 5:** Mean and standard error of normalised signal change for each saccade event type.

	PROSACCADE		ANTISACCADE	
	IPSILATERAL (μ±σ)	CONTRALATERAL (μ±σ)	IPSILATERAL (μ±σ)	CONTRALATERAL (μ±σ)
SC	0.0813±0.0250	0.1118 ±0.0199	0.1056±0.0182	0.0969 ±0.0174

### Collicular activation during the preparation and execution of pro-saccades and anti-saccades

**Fig 9:**
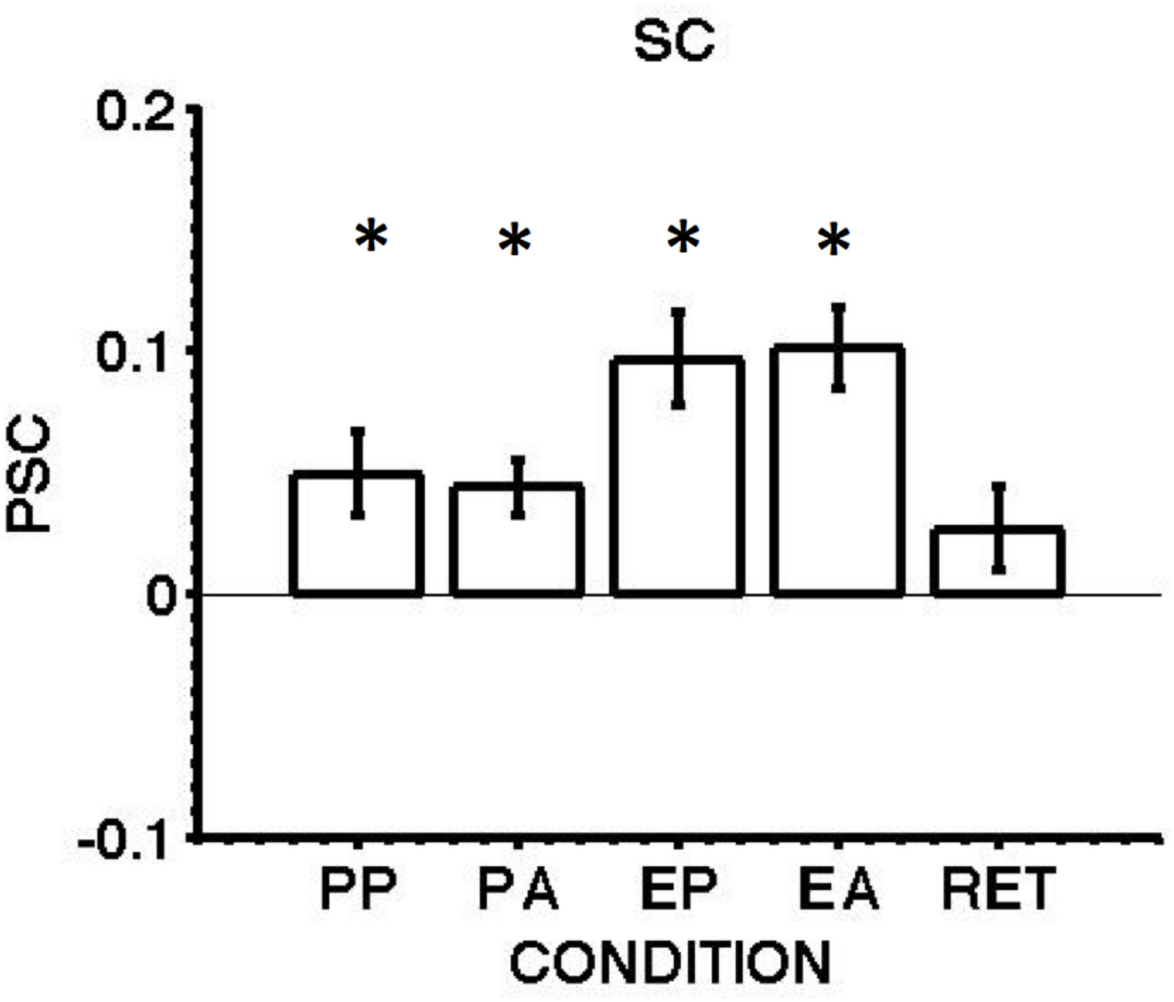
BOLD responses, averaged across 12 hemispheres from 6 subjects for preparation and execution of pro-saccades and anti-saccades, in the superior colliculus. Key as for Fig 4.

Experiment 2 aimed to examine whether anti-saccades produce greater activity than pro-saccades in the SC during the preparation and execution phases of both types of saccades. Fig 9 shows the mean saccade response magnitude for pro-saccades, anti-saccades and return saccades, while the means and standard errors of the normalised percentage signal change for each of these conditions is reported in Table 6. The responses related to pro-saccades and anti-saccades are shown separately for the preparation phase (PP, PA) and the execution phase (EP, EA) although as noted this distinction is not appropriate for the centripetal return saccades (RET). The image from the eye camera was continuously monitored during scanning and very few direction errors were observed.

**Table 6:** Mean and standard error of normalised signal change for each saccade event type.

	PREPARATION		EXECUTION		RETURN
	PROSACCADE (μ±σ)	ANTISACCADE (μ±σ)	PROSACCADE (μ±σ)	ANTISACCADE (μ±σ)	(μ±σ)
SC	0.0492 ±0.0172	0.0437 ±0.0114	0.0966 ±0.0191	0.1013 ±0.0169	0.0268 ±0.0173

The type of saccade (pro-saccade, anti-saccade) did not modulate the level of hemodynamic activity in the SC [F_(1,11)_ = 0.001, n.s.]. However, the hemodynamic response increased with saccade phase (preparation, execution) [F_(1,11)_ = 8.087, p < 0.012]. Preparing or executing any saccade equally affected the type of saccade generated [F_(1,11)_ = 0.173, n.s.]. P*ost hoc* contrasts revealed that the preparatory phase of a saccade produced a significant increase compared to baseline in the SC BOLD response for both pro-saccades (t_(17)_ = 2.85, p = 0.01) and anti-saccades (t_(17)_ = 3.82, p = 0.001). As expected, a greater response was observed for saccade execution for both pro-saccades (t_(17)_ = 5.05, p < 0.001) and anti-saccades (t_(17)_ = 5.99, p < 0.001). The amplitude of the response generated in the preparatory phase of pro-saccades was almost half (50.9%) of the response produced by executing it and a similar but stronger pattern was observed for anti-saccades with the response produced for execution being 56.9% greater than the response during preparation (t_(17)_ = -3.11, p = 0.006).

The findings for pro-saccades are consistent with our previous report [27] in which we observed that the preparatory phase of pro-saccades produced an increase in the hemodynamic response within the SC, and that this response was roughly half of the response produced by the execution of a saccade. The responses observed during return saccades to the central fixation point were small and not statistically reliable (t_(17)_ = 1.55, p = n.s.), also consistent with our previous study.

### Decoding pro-saccades from anti-saccades during the preparation and execution phase in the SC

**Fig 10:**
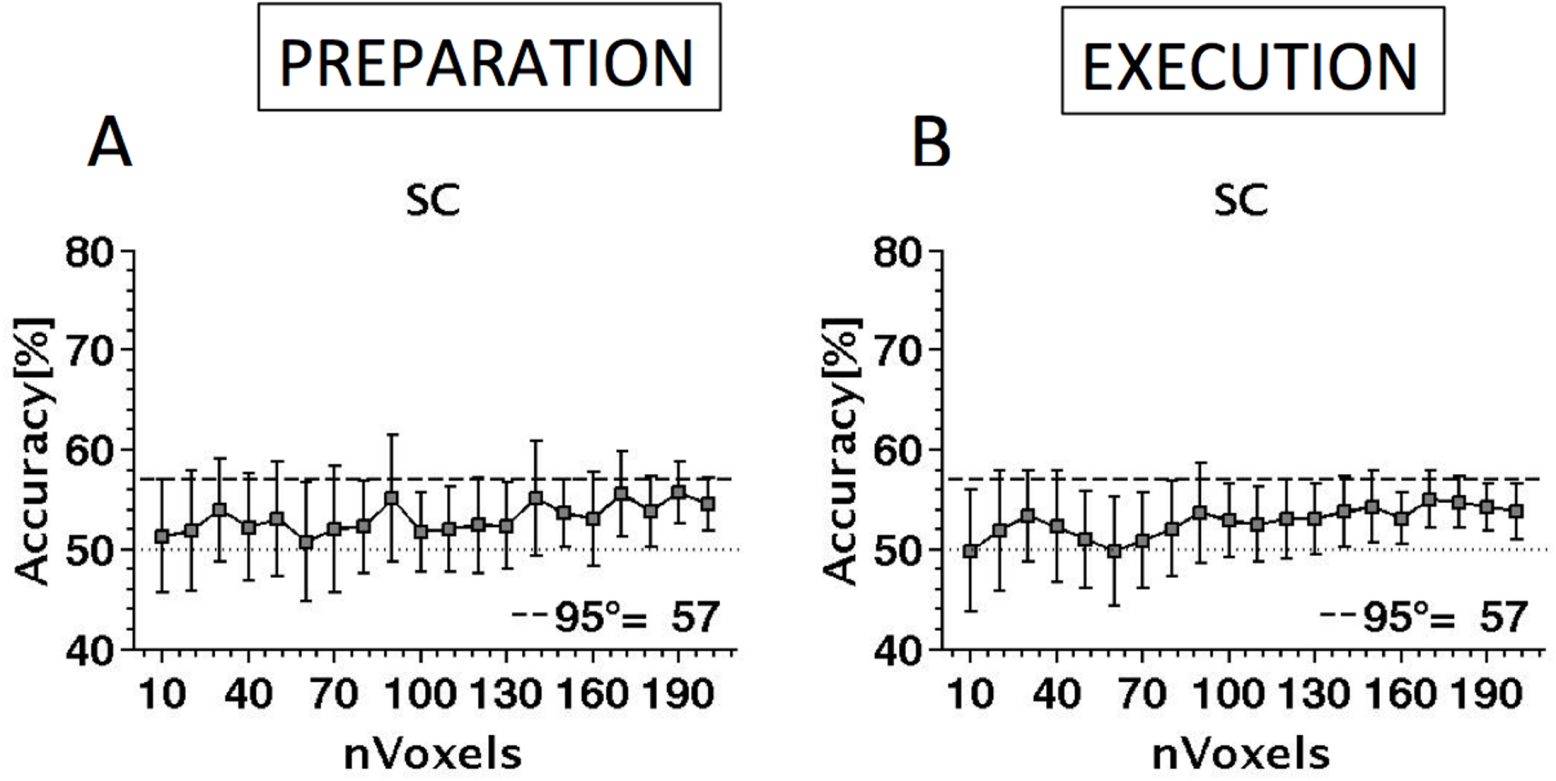
Decoding performance in the SC shown as a function of voxel sample size (number of exemplars) included in the MVPA analysis. Performance for distinguishing anti-saccade from pro-saccade is shown for the preparation phase (A) and the execution phase (B). Key as for Fig 5.

Fig 10 shows the ability of the SVM to decode (predict) which class a given stimulus belonged to as a function of the number of voxels (exemplars) included in the analysis. Because of the reduced size of the SC compared to the cortical regions analysed in Experiment1, in Experiment 2 the maximum number of voxels was 200 instead of 500. Also shown is the chance level accuracy (50%, dotted line) together with the 95^th^ percentile of the distribution of accuracies based on the permutation testing in the relevant visual area. The SVM was not able to reliably distinguish pro-saccades from anti-saccades, either during the preparation or execution of either pro- or anti- saccades. In both cases performance was numerically above chance and increased slightly with sample size, suggesting a possible difference in the representation of pro-saccades and anti-saccades in the SC, but we were not able to demonstrate such an effect clearly. The multivariate analysis might, however, be biased in favour of the cortex (Experiment 1) as the number of active samples in the DLPFC, FEF and IPS is much greater than for the SC. Although this is plausible we have shown that two classes can be significantly decoded even with <200 voxels [35] and so the current analysis should have enough statistical power to decode pro-saccades and anti-saccades in the SC.

## Discussion: SC Activity

Experiment 2 was performed to examine the SC response during the planning and execution of pro- and anti-saccades. An increase in collicular activity was observed during the preparatory phase of pro and anti-saccades, but this response was not modulated by the type of upcoming saccade. The absence of differential activation contrasts with the finding of greater BOLD response for anti-saccades in the cortex observed in earlier studies [16,17,37] and in our Exp 1. Executing a saccade produced a response with roughly twice the amplitude of that observed in the preparation phase [27] but again there was no difference in response for pro- and anti-saccades. The MVPA classifier was unable to decode pro-saccade from anti-saccade, either during the preparation phase or the execution phase. Indeed, irrespective of how many voxels were analysed, the highest accuracy in predicting the saccade type was never higher than the 95^th^ percentile from permutation testing.

The use of a delayed saccade paradigm, in which response type but not direction was cued, may have contributed to the classifier being unable to decode pro and anti saccade activity during the preparatory phase. Signals from the prefrontal cortex to the SC may be sent to the SC after target onset and may not be maintained during the cueing period so the pattern of preparatory activity is similar. For saccade execution differences in activity would be expected in the SC and it is possible that MVPA is unable to detect it. Recent work by Johnston and Everling [22] indicates that the cortical drive to saccade neurons is reduced for anti-saccades which produces a reduction in ipsilateral SC activity. This would result in reduced activity rather than a different pattern of activity that would not be detected by MVPA.

## General Discussion

The preparation to make a particular type of response and the readiness to act (or ‘preparatory-set’) has been investigated in studies that have applied functional imaging (fMRI) to examine the BOLD response in cortical regions while participants prepare to make either a pro-saccade or an anti-saccade [16,17,37]. These studies have demonstrated a role for the human frontal eye fields in preparatory set, along with the dorsolateral prefrontal cortex and intraparietal sulcus. We [27] recently investigated pre-response preparatory activity in the human superior colliculus using fMRI and a delayed pro-saccade paradigm. Saccade direction was pre-cued and an increase in the collicular BOLD response was observed in the instruction pre-response period that was attributed to preparatory activity in saccade-related neurons such as those found in the intermediate layers (See: [19,21][40] for reviews). The present study extended this by investigating preparatory activity associated with the planning of a specific type of response (pro-saccade or anti-saccade) in both the cortex and superior colliculus. We implemented the paradigm used by DeSouza et al. [17]where the type of forthcoming response (pro or anti) was specified by a symbolic cue, but saccade direction was not specified. Consistent with the findings of DeSouza and colleagues an elevated BOLD response was reliably elicited, for anti-saccades compared to pro-saccades, in the cortical oculomotor regions of interest (FEF, DLPFC and IPS). Furthermore, MVPA was able to reliably classify the type of response in the IPS and also the type of response that was executed (pro or anti) in the DLPFC, FEF and IPS. The ability to decode activity associated with voluntary and stimulus-elicited saccades in the intraparietal sulcus and precentral sulcus is consistent with a recent report by Bender and colleagues [41]. They showed that the classifier could distinguish saccade direction in the IPS equally well for both forms of saccades, and in the PCS (regarded as the human homologue of the FEF) accuracy was increased for internally generated saccades consistent with this region having a greater role in volitional control

The results for the superior colliculus were different and a generalised increase in BOLD response was observed during the preparatory pre-response period. The increase in SC response during the pre-response period is consistent with evidence of preparatory activity previously reported for delayed pro-saccades [27]. In the present study an increase in collicular response was observed during the preparation of pro- and anti- saccades, but this activity was not modulated by the type of upcoming response specified by the pre-cue. The MVPA analysis was also not able to distinguish between pro- and anti- saccades during the preparatory or saccade execution phase in the SC. In contrast to the cortical response the superior colliculus appears to have a more generalised role in the preparation to make a future action, but the pre-programming of cognitive processes relating to the particular type of response would appear to be mediated by the cortical regions.

A distinction can be made between different types of preparatory activity: one is associated with the generalised preparation to make any response, another is the pre-programming of a saccade to a pre-specified spatial location and a third is the planning of a particular type of response that involves different cognitive processes (such as response inhibition and vector inversion in the case of anti-saccades). Curtis and Connolly [16] reported an elevated BOLD response in the pre-central sulcus and intraparietal sulcus during the preparatory instruction period for anti-saccades, that was further elevated when saccade metrics were specified. This was attributed to increased activity of neurons involved in saccade planning including a non-spatial anticipation of a future action and also saccade-related neurons involved in the planning of a specific response towards a specific saccade goal. The greater activity in the FEF, IPS and DLPFC we observed on anti-saccade trials in the pre-response period is consistent with these regions having a role in implementing task-specific instructions in addition to a generalised preparation to making a future action. Saccade direction was not specified in the preparatory period so activity associated with the saccade goal would not be included here. We also observed evidence of preparatory activity in the human SC, but unlike that in the cortex this was not modulated by response type. Thus we would conclude that although the colliculus has a role in the preparation to make a future response it does not appear to be involved in the preparation to implement task instructions; this appears to be mediated by the cortex.

The lack of differentiation in the collicular BOLD response contrasts with neurophysiological evidence that neural activity in the primate SC is modulated by the instruction to make a pro- or anti-saccade in the absence of information about forthcoming saccade location. Everling et al. [26] showed increased activity in collicular fixation neurons and decreased activity in the saccade-related neurons during the instruction phase to make an anti-saccade. The pre-saccadic visual response was reduced for anti saccades and a weaker motor burst observed during saccade initiation. It was proposed that the saccade-related neural discharge may be insufficient on its own to trigger an anti-saccade which may require additional inputs from FEF and SEF to the brainstem saccade generator. In our results the collicular BOLD signal was not modulated on anti-saccade trials, as was observed for the cortex, and MVPA analysis did not reveal differences in the underlying pattern of neural activity for the two types of response. Discrepancies between neural spike rates in neurophysiological studies and the haemodynamic response in human imaging studies have been noted previously and may potentially arise because the BOLD signal is sensitive to synaptic activity associated with response inhibition [27,42,43]. The increased BOLD response in the SC, during the pre-response phase for both pro and anti-saccades, is consistent with a generalised role of collicular neurons in response preparation [27] rather than in the preparation of the specific response type (pro or anti) with unspecified direction. The colliculus appears not to have a role in the preparation to make a specific type of response that may depend on signals originating in the cortex that may provide an additional input to the brainstem for the initiation of an anti-saccade. The cortical activation of collicular saccade-related neurons may be reduced for anti-saccades in order to reduce the reflexive response triggered by the target, rather than enhancing fixation activity as previously thought [44]. Thus the pattern of collicular activity may be similar during the preparation of both types of response. We cannot discount the possibility however that fMRI was not sensitive enough to reveal a modulation of the collicular response during the preparation of pro or anti saccades.

Consistent with our earlier report [27] the SC response during saccade execution was bilateral and was not strongly lateralised for the direction of the upcoming saccade. Furlan et al. [27] used a contralaterality index to compare activity in the left and right SC and were able to show an association between contralateral activity and saccade direction, although this does not appear to be strongly lateralised in the haemodynamic response. This bilateral increase in BOLD may reflect the nature of the BOLD signal itself, although it is at least possible that the human SC is less lateralised than is the monkey (see: 32 for a discussion on this point)

Finally, the cortical and collicular hemodynamic activity for return (centripetal) saccades was reduced compared to that associated with outward (centrifugal) pro- and anti- saccades. The reduced activity for return saccades is consistent with earlier findings [27,38,45]. However, although Krebs and colleagues [38] observed a similar reduction in activity for return saccades in the SC this was not found in the cortical regions. They suggested that return saccades maybe a relatively automatic response and as such largely mediated by the SC with concomitant reduced levels of cortical involvement. In our study a similar reduction in response was observed in the cortex and colliculus for return saccades, implying a similar level of involvement in the generation of return saccades. There are substantial differences between their task and ours. The task used by Krebs and colleagues involved endogenous cues and additional higher-level cognitive functions such as memory load associated with frontal lobe functions [46]. Our task was simpler and less cognitively demanding and this might have enabled us to isolate the response associated with return saccades. The pattern of responses created by return saccades was similar in the SC and in the cortical regions, which suggests that return saccades are mediated not only in the SC but also in the cortical regions, but the overall level of processing required is reduced compared to that required for the planning and execution of outward saccades.

## Conclusions

The present study has revealed a preparatory response in the human cortical oculomotor regions and in the superior colliculus. The response in the FEF, IPS and DLPFC was modulated by the type of upcoming response and was greater for anti-saccades than pro-saccades. MVPA was able to reliably classify the type of response executed in all three regions and in the IPS for response preparation. The BOLD response in the superior colliculus increased during the instruction pre-response phase for both pro-saccades and for anti-saccades that may reflect a generalised non-spatial preparation to make a future action. The collicular preparatory response was not sensitive to the type of response being prepared and MVPA was not able to classify the type of response type. Thus, the SC shows some preparatory response that may reflect response preparation but the cognitive set relating to a specific type of response may depend on the cortex.
